# ABO and Rhesus blood group markers as predictors in colorectal cancer: A prospective observational study

**DOI:** 10.1097/MD.0000000000036256

**Published:** 2023-11-24

**Authors:** Gowhar Rashid, Gulzar A. Bhat, Tahseen Bilal Rather, Kulsum Akhtar, Ishrat Parveiz, Syed Nisar Ahmad, Malik Tariq Rasool, Farooq Ahmad Jan, Mohanad Diab, Wael Hafez, Syed Mudassar

**Affiliations:** a Department of Clinical Biochemistry, SKIMS, Srinagar, India; b Department of Amity Medical School, Amity University, Manesar, Haryana, India; c Multidisciplinary Research Unit, Sher-I-Kashmir Institute of Medical Sciences (SKIMS), Srinagar, India; d Department of Medical Oncology, SKIMS, Srinagar, India; e Department of Radiation Oncology, SKIMS, Srinagar, India; f Department of Hospital Administration, SKIMS, Srinagar, India; g Burjeel Hospital, Abu Dhabi, United Arab Emirates; h Stockholm University, Karolinska, Sweden; i NMC Royal Hospital, Khalifa City, Abu Dhabi, United Arab Emirates; j The Medical Research Division, Department of Internal Medicine, The National Research Centre, Cairo, Egypt.

**Keywords:** blood group, colorectal cancer, Kashmir, prognosis, survival

## Abstract

Numerous research studies have investigated the relationship between ABO and Rhesus (Rh) blood groups and the risk of various cancers, yielding diverse findings. While these blood groups have been established as prognostic factors in some cancers, their relevance to colorectal cancer (CRC) remains uncertain. This research aims to determine the link between CRC and the ABO and Rh blood groups and explore any potential implications for disease survival. A hospital-based prospective observational study was conducted from March 2019 to March 2022 at the Sher-I-Kashmir Institute of Medical Sciences in Srinagar, India. A total of 246 patients with confirmed colorectal cancer were enrolled in the study. Our study observed that blood type B (33.74%) and Rh-positive (91.87%) blood types were the most prevalent, surpassing other blood groups. No statistically significant associations were identified between the blood groups and the studied xenobiotic-metabolizing enzyme gene variants. The study observed a heightened risk of CRC in patients with advanced cancer stages and lymphovascular invasion (*P*-value < .05). On follow-up, there were no statistically significant differences in 3-year survival rates observed between ABO and Rh blood groups. This study’s findings suggest that ABO and Rh blood groups are not associated with the risk of CRC or overall survival among CRC patients. Further clinical studies are needed to establish the precise relationship between blood groups and CRC risks, as well as their implications for the prognosis of CRC patients.

## 1. Introduction

Kashmir valley is experiencing an increase in the prevalence of gastrointestinal cancers, which are the main cause of mortality and morbidity in the region.^[1,2]^ Despite various proposed factors, including unusual food patterns, the evidence remains uncertain. Colorectal cancer (CRC) is one of the major public health concerns globally^[3]^ and is predicted to be 60% more prevalent by 2030.^[4]^ There has been a significant increase in CRC incidence and mortality in medium and high-human development index countries.^[5]^ Advances in therapeutic protocols have greatly improved treatment for CRC, but the prognosis remains poor. Several risk factors, including genetic^[6,7]^ and environmental factors^[8–10]^ have been linked to the development of CRC genesis. At present, several risk factors such as smoking, drinking alcohol, consuming red meat, eating little vegetables, being obese, having a history of cancer in the family, physical inactivity, and bowel problems have been identified to be linked with CRC.^[10,11]^

The ABO blood groups, defined by carbohydrate moieties present on the surface of red blood cells, are medically the most important blood types. They are attached to a protein backbone known as the H antigen.^[11]^ In addition to being present in red blood cells, ABO antigens are also highly expressed on the surface of epithelial cells of various organs like gastrointestinal tracts.^[11]^ Alterations in surface glycoconjugates may cause changes in many cellular processes, such as membrane signaling and intercellular adhesion, resulting in a series of events that lead to disease development, including various types of malignancies.^[12,13]^ Few laboratory studies have proposed various possible mechanisms for the observed relationships between ABO blood types and cancer, such as immunological defense against cancerous cells, intercellular adhesion, and membrane signaling.^[14–16]^ A recent study suggested a possible association between ABO blood groups and the likelihood of having CRC.^[17]^ Blood group antigens are present on the outer layer of erythrocytes and other tissues, such as epithelial cells of the gastrointestinal tract. The glycoconjugates in the ABO blood group antigens may play a role in changing intercellular adhesion, membrane signaling, and immune surveillance, which could influence tumorigenesis.^[12]^ In addition, new genome-wide association studies have reported an association between single nucleotide polymorphisms in the ABO blood group with circulating levels of tumor necrosis factor-alpha and diabetes mellitus.^[18,19]^ Further, colorectal neoplasia is associated with the inflammatory marker, tumor necrosis factor-alpha.^[20]^ In the study^[14]^ by Khalili et al, who found no significant association between blood group B and the overall risk of colon cancer. However in the other study.^[21]^ Conversely, Kashfi et al reported a significant relationship between blood group and colon cancer. Similarly, in another study, Urun et al reported that ABO/Rh (Rhesus) blood groups were significantly associated with CRC risk and that there was no relationship between K-ras status and the ABO blood group and Rh factor.^[17]^

Previous research has indicated that blood type and the RH factor may influence various aspects of human physiology and immunity. There is some evidence to suggest that blood type antigens and Rh factors may play a role in the risk of certain diseases, including colorectal cancer. Although there have been studies examining the links between blood type, the Rh factor, and CRC, there is a noticeable gap in the literature regarding colorectal cancer specifically in the Kashmir region, India. Our study aims to fill this void by providing valuable insights into a less-explored area of research. Hence, the objective of this study is to ascertain the potential influence of ABO and Rh blood groups on the risk, clinicopathological characteristics, and prognosis of colorectal cancer within indigenous communities in India.

## 2. Materials and methods

### 2.1. Study design and sample

A hospital-based prospective observational study was conducted at Sher-I-Kashmir Institute of Medical Sciences (SKIMS) Srinagar, India. Patients visiting SKIMS for treatment in the Department of medical oncology and radiation oncology, from March 2019 to March 2022, were recruited for this study. A total of 246 CRC patients were involved in the study. The sample (N = 246) was based on the number of cases of CRC among the ABO blood group and had >80% power to detect an HR of 1.2 at a significance level of 0.05. Due to Kashmir’s geographical isolation, SKIMS mostly serves patients from the local population with a common Kashmiri ethnicity. This study included participants who met the inclusion criteria, with Kashmir as their birthplace, participants should have a documented diagnosis of CRC and participants must be willing to provide informed consent to participate in the study, and excluded individuals who met the exclusion criteria, which included those suffering from any malignancy other than CRC. The recruitment of the patients was done after seeking informed consent. Participants were further categorized according to the frequency and specific genotype alleles they carried. In terms of CYP genes, individuals exhibited either a homozygous wild-type genotype or a variant genotype (the latter containing at least one or both mutant alleles). Regarding glutathione-S-transferases, the wild genotype states were indicated as GSTM1+ and GSTT1+, while the null genotype conditions were GSTM1− and GSTT1−, respectively. Furthermore, a blood sample was collected from each participant for DNA extraction. Subsequent DNA analysis encompassed genotyping for XME genes, cytochrome p450 (CYP) enzymes, and glutathione-S-transferases.

### 2.2. Assessment of ABO blood group and covariates

Patients diagnosed with CRC through histopathological diagnosis were screened for ABO and Rh blood groups. Blood samples were collected from each patient during their outpatient visit. ABO and Rh blood groups were determined using slide agglutination testing. The frequency and distribution of each phenotype (A, B, AB, and O) along with their Rh status were recorded. We used a questionnaire to record participants’ socio-demographic status, including the history of their smoking status (current, past, or never) and the history of any disease conditions (such as diabetes and hypertension).

### 2.3. Identification of outcome events

We follow up on CRC patients over the phone monthly to record their survival outcomes. The OS was calculated from the surgery until death from any cause or until the last follow-up (March 2022).

### 2.4. Statistical analysis

Baseline characteristics were presented as frequency and percentages. Categorical data was analyzed using the Chi-square/Fisher exact test to detect differences. The time-to-event analysis was presented with the Kaplan–Meier Curve. Log-rank test and Cox regression were used to demonstrate the differences in each categorical group. *A P*-value of <.05 was considered statistically significant. All the statistical analysis was conducted using STATA software, version 16 (STATA 16 Corp., College Station, TX), and statistical package for the social sciences (SPSS) software, version 26.

## 3. Results

In the ABO blood group system, 50 individuals (20.33%) have blood type A, 83 (33.74%) have blood type B, and 35 (14.23%) have the AB blood type. The most prevalent blood type is O, with 78 (31.71%) falling into this category. Regarding the Rh blood group system, 226 (91.87%) are Rh+, indicating the presence of the Rh antigen on their red blood cells, while 20 (8.13%) are Rh−, signifying the absence of this antigen. Among the study sample, antigen B was higher than other groups, with a percentage of 33.74% (83 out of 246) followed by antigen O (31.71%). Rh-positive subjects showed the maximum presentation (91.9%) (Table 1).

**Table 1:** The ABO and Rhesus blood groups among the enrolled patients.

Variable		n (%)
AB blood groups	A	50 (20.33%)
	B	83 (33.74%)
	AB	35 (14.23%)
	O	78 (31.71%)
Rhesus blood groups	Rh+	226 (91.87%)
	Rh-	20 (8.13%)

The demographic and clinicopathological attributes of all subjects grouped by ABO and Rh blood groups were shown in Table 2. In the “Age Group” category, blood group distribution varies significantly (*P* = .008) across age groups. For instance, in the 0 to 25 age group, blood type A is the most common (63.64%), and the majority are Rh+ (90.91%). The table shows that as age increases, the distribution of blood groups also changes. The “Gender” variable shows no significant association between blood groups and gender, with *P*-values >.05. The same applies to “Tumor Differentiation,” where the distribution of blood groups is not significantly related to how well-differentiated the tumor is. Conversely, in the “Diagnosis” category, there is a significant association (*P* = .049) between blood groups and cancer type. For example, a significant proportion of patients with colon cancer have blood type A (28 out of 149), and a considerable majority of them are Rh+ (140 out of 148). The “Node Involvement” variable shows a borderline significant association (*P* = .151) where patients with blood type O seem to have a higher incidence of node involvement. In the “Number of Nodes Involved” category, significant associations are found for some variables. Patients with blood type A have a higher number of nodes involved (*P* = .007). In the “LVI” category, there is a significant association with *P* = .048, indicating that patients with blood type B have a higher incidence of lymphovascular invasion (LVI). The table further extends the analysis to other variables, such as “PNI,” “TALNR,” “Necrosis,” “Family History of Any Cancer,” and clinical parameters like “CEA,” “Hb,” and “PLT.” In most cases, there is no significant association between these variables and blood groups, as indicated by *P*-values >.05. On analysis, we observed a statistically significant but differential CRC risk among different age groups harboring a specific blood group (*P* = .008) and several nodes involved (*P* = .007). Similarly, the positive Rh factor was significantly distributed among the 2 main types of CRC (*P* = .049) and LVI (*P* = .048). However, there was no difference among subjects harboring these blood groups and other factors like gender, site of a tumor, tumor differentiation, node involvement, staging, invasion depth, perineural invasion (PNI), tumor associated lymph node response family history of cancer, and other clinical parameters (*P* > .05) (Table 2).

**Table 2:** The correlation between demographic and clinicopathological characteristics of the ABO blood group and Rh factors among the enrolled patients.

Variable	ABO blood group					Rhesus blood group		
	A	B	AB	O	*P*-value	Rh+	Rh-	*P*-value
*Age group*								
0–25	7 (63.64)	3 (27.27)	0 (0.00)	1 (9.09)	**.008**	10 (90.91)	1 (9.09)	.947
26–50	10 (13.16)	31 (40.79)	10 (13.16)	25 (32.89)		69 (90.79)	7 (9.21)	
51–75	33 (22.00)	45 (30.00)	22 (14.67)	50 (33.33)		139 (92.67)	11 (7.33)	
≥75	0 (0)	4 (44.44)	3 (33.33)	9 (22.22)		8 (88.69)	1 (11.11)	
*Gender*								
Male	32 (21.48)	44 (29.53)	49 (16.72)	49 (32.89)	.343	141 (94.63)	8 (5.37)	.051
Female	18 (18.56)	39 (40.21)	11 (11.34)	29 (29.90)		85 (87.63)	12 (87.63)	
*Diagnosis*								
Ca colon	28 (18.92)	46 (31.08)	23 (15.54)	51 (34.46)	.479	140 (94.59)	8 (5.41)	**.049**
Ca rectum	22 (22.45)	37 (37.76)	12 (12.54)	27 (27.55)		86 (87.76)	12 (12.54)	
*Site of tumor*								
Colon	28 (20.29)	40 (28.99)	22 (15.94)	48 (34.78)	.552	130 (94.20)	8 (5.80)	.308
Rectum	21 (21.65)	38 (39.18)	12 (12.37)	26 (26.80)		86 (88.66)	11 (11.34)	
Rectosigmoid	1 (9.09)	5 (45.45)	1 (9.09)	4 (36.36)		10 (90.91)	1 (90.91)	
*Tumor differentiation*								
Well differentiated	10 (19.61)	19 (37.25)	6 (11.76)	16 (31.37)	.680	46 (90.20)	5 (9.80)	.810
Moderately differentiated	32 (19.88)	50 (31.06)	27 (16.77)	52 (32.30)		148 (91.93)	13 (8.07)	
Poorly differentiated	8 (23.53)	14 (41.81)	2 (5.88)	10 (29.41)		32 (94.12)	2 (5.88)	
*Node involvement*								
Yes	7 (14.58)	12 (25.00)	8 (16.67)	21 (43.75)	.151	46 (95.83)	2 (4.17)	.263
No	43 (21.72)	71 (35.86)	27 (13.64)	57 (28.79)		180 (90.91)	18 (9.09)	
*Number of nodes involved*								
0	43 (21.50)	72 (36)	27 (13.50)	58 (29)	**.007**	182 (91)	18 (9.00)	.582
1 & 2	2 (8.70)	3 (13.04)	8 (34.78)	10 (43.48)		22 (95.65)	1 (4.35)	
>2	5 (21.74)	8 (34.78)	0 (0.00)	10 (43.48)		22 (95.65)	1 (4.35)	
*Duke stage*								
A	17 (21.52)	26 (32.91)	14 (17.72)	22 (27.85)	.09	73 (92.41)	6 (7.59)	.492
B	20 (19.80)	41 (40.59)	11 (10.89)	29 (28.71)		90 (89.11)	11 (10.89)	
C	4 (12.90	5 (16.13)	8 (25.81)	14 (45.16)		29 (93.55)	2 (6.45)	
D	2 (13.33)	7 (46.67)	0 (0.00)	6 (40.00)		15 (100)	0 (0.00)	
*TNM stage*								
I	11 (21.57)	18 (35.29)	10 (19.61)	12 (23.53)	.293	46 (90.20)	5 (9.80)	.848
II	14 (15.22)	37 (40.22)	12 (13.04)	29 (31.52)		86 (93.48)	6 (6.52)	
III	7 (20.00)	6 (17.14)	7 (20)	15 (42.86)		32 (91.43)	3 (8.57)	
IV	11 (22.92)	18 (37.50)	4 (8.33)	15 (31.25)		43 (89.58)	5 (10.42)	
*Invasion depth*								
T1	1 (10.00)	2 (20.00)	1 (10.00)	6 (60.00)	.595	9 (90.00)	1 (10.00)	.932
T2	15 (20.27)	27 (36.49)	14 (18.92)	18 (24.32)		69 (93.24)	5 (6.76)	
T3	25 (19.38)	44 (34.11)	17 (13.18)	43 (33.33)		117 (90.70)	12 (9.30)	
T4	2 (15.38)	6 (46.15)	43 (33.33)	4 (30.77)		12 (92.31)	1 (7.69)	
*LVI*								
Present	22 (18.49)	41 (34.45)	18 (15.13)	38 (31.93)	.989	113 (94.96)	6 (5.04)	**.048**
Absent	21 (19.63)	38 (35.51)	15 (14.02)	33 (30.84)		94 (87.85)	13 (12.15)	
*PNI*								
Present	6 (23.08)	12 (46.15)	2 (7.69)	6 (23.08)	.404	24 (92.31)	2 (7.69)	0.889
Absent	37 (18.50)	67 (33.50)	31 (15.50)	65 (32.50)		183 (91.50)	17 (8.50)	
*TALNR*								
Present	35 (18.32)	67 (35.08)	31 (16.23)	58 (30.37)	.392	174 (91.10)	17 (8.90)	.532
Absent	8 (22.86)	12 (34.29)	2 (5.71)	13 (37.14)		33 (94.29)	2 (5.71)	
*Necrosis*								.608
Present	11 (22.45)	15 (30.61)	4 (8.16)	19 (38.78)	.311	44 (89.80)	5 (10.20)	
Absent	32 (18.08)	64 (36.16)	29 (16.38)	52 (29.38)		163 (92.09)	14 (7.91)	
*Family history of any cancer*								
Yes	10 (18.52)	19 (35.19)	10 (18.52)	15 (27.78)	.985	48 (92.31)	4 (7.69)	.897
No	74 (18.88)	145 (36.99)	65 (16.58)	108 (27.55)		178 (91.75)	16 (8.25)	
*CEA*								
<3	20 (20.83)	33 (34.38)	13 (13.54)	30 (31.25)	.993	89 (92.71)	7 (7.29)	.735
≥3	26 (20.16	43 (33.33)	19 (14.73)	41 (31.56)		118 (91.47)	11 (8.53)	
*Hb*								
<13.5 g/dL	56 (19.11)	108 (36.86)	49 (16.72)	80 (27.30)	.996	116 (90.22)	18 (9.78)	.102
≥13.5 g/dL	28 (18.30)	56 (36.60)	26 (16.99)	43 (28.10)		60 (96.77)	2 (3.23)	
*PLT*								
<150	19 (21.59)	31 (35.23)	13 (14.77	25 (28.41)	.846	46 (92.00)	4 (8.00)	.970
≥150	65 (18.16)	133 (37.15)	62 (17.32)	98 (27.37)		180 (91.84)	16 (8.16)	
*Calcium*								
<8.8	26 (17.45)	58 (38.93)	24 (16.11)	41 (27.52)	.651	48 (88.89)	6 (11.11)	.348
≥8.8	34 (18.68)	62 (34.07)	38 (20.88)	48 (26.37)		72 (93.51)	5 (6.49)	

The bold values in Table 2 indicate statistical significance, as the p-value is equal to or less than 0.05.

CEA = carcinoembryonic antigen, Hb = hemoglobin, LVI = lymphovascular invasion, PNI = perineural invasion, PLT = platelets, TALNR = tumor associated lymph-node response.

The association of a particular blood group towards CRC development in subjects harboring a specific genotype is presented in Table 3. Considering “CYP2A13,” there are 35 (21.60%) with the “Wild” genotype, 13 (17.33%) with the “Heterozygous” genotype, and 2 (22.22%) with the “Mutant” genotype. Notably, in the “CYP2A13” section, a P-value of .100 suggests no statistically significant association between cytochrome P450 2A13 (CYP2A13) genotypes between the blood group types. Furthermore, it reports the counts of individuals who are either Rh+ or Rh− for blood type (e.g., in the “CYP2A13” section, 146 individuals are Rh+ [90.12%], and 16 individuals are Rh− [9.88%]). We could not find any significant distribution of the ABO blood group among subjects and hence no correlation between the 2 groups in predicting the CRC risk (*P* > .05) (Table 3).

**Table 3:** Correlation between blood group typing and various genetic variants of XMEs.

Genotype	A	B	AB	O	*P*-value	*P*-value
							Rh+	Rh−
*CYP2A13*								
Wild	35 (21.60)	57 (35.19)	19 (11.73)	51 (31.48)	.767	146 (90.12)	16 (9.88)	.151
Heterozygous	13 (17.33)	23 (30.67)	15 (20.00)	24 (32.00)		73 (97.33)	2 (2.67)	
Mutant	2 (22.22)	3 (33.33)	1 (11.11)	3 (33.33)		7 (77.78)	2 (22.22)	
*CYP2A6a*								
Wild	49 (22.90)	73 (34.11)	30 (14.02)	62 (28.97)	.100	196 (91.59)	18 (8.41)	.313
Heterozygous	1 (3.57)	8 (28.57)	5 (17.86)	14 (50.00)		27 (96.43)	1 (3.57)	
Mutant	0 (0.00)	2 (50.00)	0 (0.00)	2 (50.00)		3 (75.00)	1 (25.00)	
*CYP26b*								
Wild	43 (22.39)	58 (30.20)	23 (11.97)	68 (35.41)	.110	175 (91.15)	17 (8.85)	.623
Heterozygous	7 (14.89)	24 (51.06)	8 (17.02)	8 (17.02)		44 (93.62)	3 (6.38)	
Mutant	1 (14.28)	1 (14.28)	3 (42.85)	2 (28.57)		7 (100.00)	0 (0.00)	
*CYP26c*								
Wild	43 (21.5)	61 (30.5)	30 (15.00)	66 (33.00)	.209	183 (91.50)	17 (8.50)	.659
Heterozygous	7 (18.91)	13 (35.13)	5 (13.51)	12 (32.43)		34 (91.89)	3 (8.11)	
Mutant	3 (33.33)	4 (44.44)	1 (11.11)	1 (11.11)		9 (100.00)	0 (0.00)	
*GSTT1*								
Present	39 (20.42)	65 (34.03)	27 (14.14)	60 (31.41)	.997	176 (91.87)	15 (7.85)	.767
Absent	11 (20)	18 (32.73)	8 (14.55)	18 (32.73)		50 (90.91)	5 (9.09)	
*GSTM1*								
Present	31 (21.68)	45 (31.47)	23 (16.08)	44 (30.77)	.625	15 (10.42)	129 (89.58)	.119
Absent	19 (18.45)	38 (36.89)	12 (11.65)	34 (33.01)		5 (4.90)	97 (95.10)	

CYP2A13 = cytochrome P450 2A13; CYP2A6a = CYP2A6 gene or cytochrome P450 2A6 enzyme; CYP26b = cytochrome P450 26B1; CYP26c = gene or enzyme within the cytochrome P450 family; GSTT1 = glutathione S-transferase theta 1; XME = xenobiotic-metabolizing enzyme.

Three of the 7 indicators of the CRC patient’s mortality were statistically significant. The patients with a stage of t3 or t4 have a higher hazard ratio (crude HR: 2.32; 95%CI: 1.09–4.95, *P*-value = .028) than those with lower stages. In addition, the patients with lymphovascular invasion and PNI have higher hazard ratios (crude HR: 2.67, 0.032; *P*-value < .05, respectively) than those with no. No significant predictor of the CRC patient’s mortality was detected when adjusting to tumor t-stage, nodes, tumor grade, PNI, LVI, ABO, and Rh blood groups (Table 4).

**Table 4:** Univariate and multivariate Cox proportional hazards regression for overall survival (N = 175).

Variables	Category	Univariate			Multivariate		
		Crude HR	95%CI	*P*-value	Adjusted HR	95%CI	*P*-value
T stage	Pt1 + pt2	1.00		**.028**	1.00		.42
	Pt3 + pt4	2.32	1.09–4.95		1.71	0.45–6.42	
Nodes	N0	1.00		**.67**	1.00		.48
	N1–3	1.16	0.56–2.42		0.68	0.23–1.96	
Grade	WD	1.00			1.00		
	MD	0.91	0.20–3.98	.9	0.96	0.20–4.63	.96
	PD	3.93	0.77–14.9	.1	3.65	0.59–22.43	.16
PNI	Absent	1.00		**.032**	1.00		.37
	Present	2.28	1.07–4.87		0.59	0.19–1.84	
LVI	Absent	1.00		**.032**	1.00		.24
	Present	2.67	1.08–6.6		1.95	0.63–5.9	
ABO	A	1.00			1.00		
	B	1.27	0.43–3.7	.65	2.24	0.69–7.23	.17
	AB	1.16	0.33–4	.81	2.06	0.53–8.01	.29
	O	1.09	0.37–3.22	.87	1.64	0.50–5.31	.40
Rhesus	RH+	1.00		.53	1.00		.28
	RH−	1.46	0.44–4.84		2.07	0.54–7.81	

The first category has been regarded as the reference category (Reference = 1.00). The bold values in Table 4 indicate statistical significance, as the p-value is equal to or less than 0.05.

**Figure 1.**
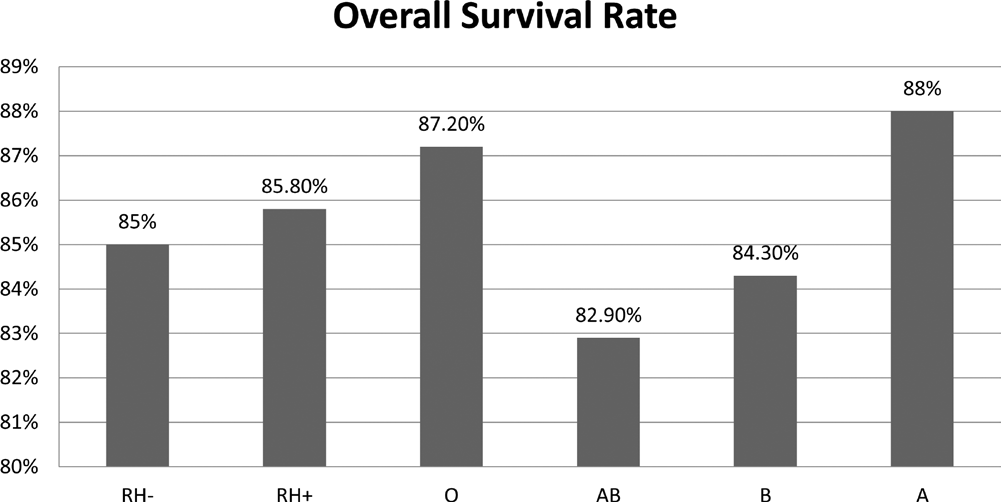
Distribution of patients and 3-year survival rates by ABO and Rh blood types.

**Figure 2.**
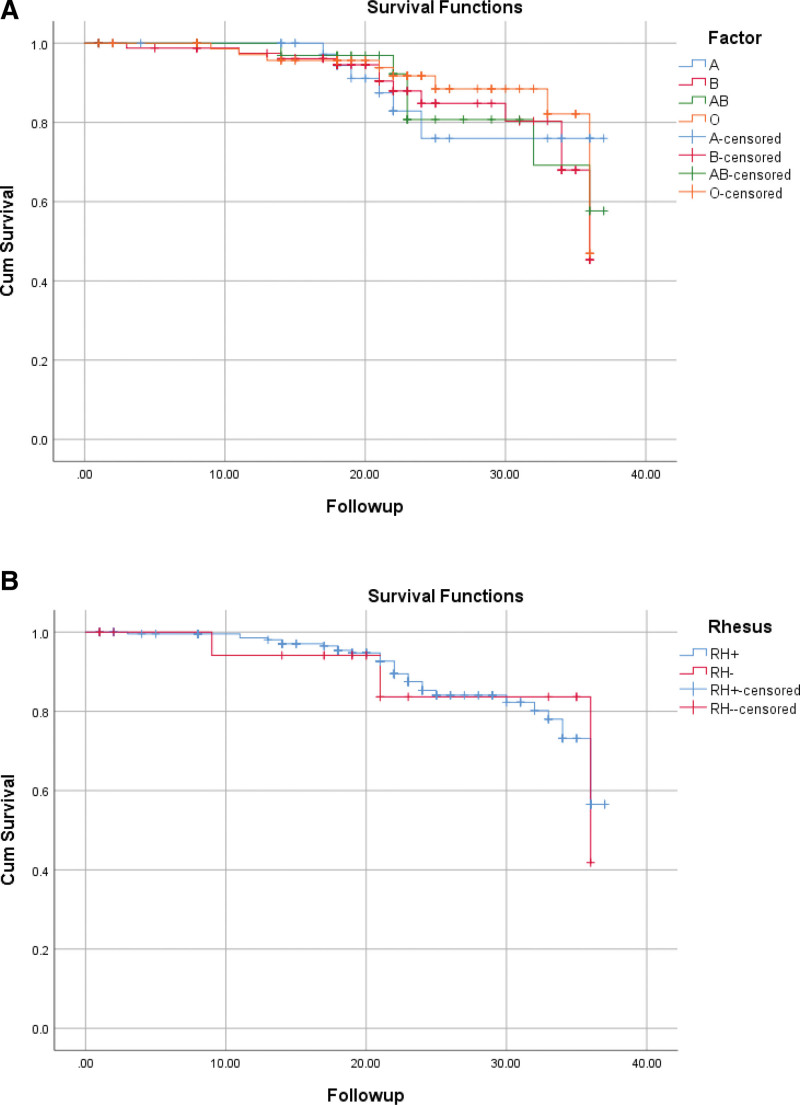
Overall survival of 246 CRC patients as per blood type. (A) ABO blood group, (B) Rh factor.

CRC patients with blood groups A, B, AB, and O had a 3-year overall survival rate of 88%, 84.30%, 82.91%, and 87.20%, respectively. Nonetheless, there is no statistically significant difference between the ABO and Rh groups regarding the overall survival rate (Fig. 1). An overview of different ABO blood groups and Rh factors for overall survival by the Kaplan–Meier curves is presented in Fig. 2.

## 4. Discussion

In our study, we investigated the distribution of various blood antigens, particularly ABO and Rh factors, among a sample of individuals and their potential associations with CRC risk and patient outcomes. We found several interesting patterns and correlations within our study sample. Firstly, we observed that antigen B was the most prevalent among our study sample, with 33.74% of the individuals having this blood type. This was followed closely by antigen O, which was found in 31.7% of the individuals. Additionally, a significant majority of the subjects were Rh-positive (91.9%). These findings indicate a notable distribution of blood types among our study population. Upon analyzing the data, we found a statistically significant but differential risk of CRC among different age groups with specific blood types. This suggests that age and blood group may have a combined effect on CRC risk. Similarly, the positive Rh factor was significantly associated with 2 main types of CRC and LVI. However, no significant differences were observed among subjects harboring these blood groups and various other factors like gender, tumor location, tumor differentiation, nodal involvement, staging, invasion depth, PNI, tumor associated lymph node response family history of cancer, and other clinical parameters. We could not establish a significant correlation between the ABO blood group and CRC risk. This indicates that while blood type may influence CRC risk, it does not appear to be a strong independent predictor. In terms of patient outcomes, our study found that several indicators were statistically significant in predicting CRC patient mortality. Patients with advanced stages of cancer (t3 or t4) had a higher hazard ratio, indicating a poorer prognosis. Similarly, the presence of LVI and PNI was associated with higher hazard ratios, suggesting a worse outcome for patients with these characteristics. However, when adjusting for various factors like tumor stage, nodal involvement, tumor grade, PNI, LVI, ABO blood group, and Rh factor, we did not identify any single significant predictor of CRC patient mortality. Regarding overall survival rates, we observed that individuals with blood groups A, B, AB, and O had varying survival rates. Nevertheless, there was no statistically significant difference in overall survival between the ABO and Rh groups, suggesting that blood type may not be a strong determinant of patient survival.

Over the last decade, many clinical studies have been conducted to investigate the association between certain types of cancer and the ABO and Rh blood groups. The hypothesis is that the ABO blood type could be a potential indicator for various malignancies, as previous research suggests a potential relationship between the ABO blood group and cancer survival and prognosis.^[12,13]^ Red blood cells and other organs, including cells in the gastrointestinal system, express human blood group antigens on their surfaces. Tumorigenesis may be influenced by the presence of these glycoconjugates due to their potential to alter intercellular adhesion, membrane signaling, and immune surveillance.^[14]^ Furthermore, recent genome-wide association studies have linked single nucleotide polymorphisms at the ABO blood locus to serum levels of circulating TNF-α, where TNF-α is an inflammatory marker connected to colorectal neoplasia.^[14]^ One another study suggested an association between ABO blood groups and the likelihood of having CRC.^[17]^ Furthermore, recent genome-wide association studies have revealed a connection between specific single nucleotide polymorphisms in the ABO blood group and the levels of tumor necrosis factor-alpha and diabetes mellitus in circulation.^[18,19]^ However, in this study, univariate and multivariate regression analysis did not reveal a significant association between the ABO and Rh blood groups with survival and risk of colorectal cancer. This is in line with the findings of previous studies that also reported an insignificant association of the ABO blood group with CRC risk.^[14]^

In disagreement with our findings, several studies have shown an association between the ABO blood group and CRC risk. For instance, Abdullah Al-Sawat et al found a statistically significant link between colon cancer and blood groups of AB and non-O when compared to the O blood group.^[15]^ Other studies have also demonstrated that blood type AB is a sufficient risk factor for individuals with colon cancer.^[16]^ Additionally, large research found that individuals with blood type B had a statistically significant decreased risk of gastrointestinal malignancies, but those with blood type AB had a higher risk of liver cancer.^[22]^

An earlier study also showed poorer 3-year survival rates for the AB blood group.^[16]^ Another study by Yang et al reported that patients with blood type O had considerably lower overall survival rates than those with non-O blood groups, according to research on esophageal cancer.^[23,24]^

Regarding the clinical and pathological parameters, except for the LVI and nodes, we did not find any statistically significant association of ABO with CRC as reported by other studies other than colorectal malignancies. Furthermore, our univariate and multivariate analysis results showed a statistically significant association between the ABO blood group and Rh with stage, PNI, and LVI. Stage, nodes, PNI, and LVI are all known to increase the risk of colorectal cancer and reduce survival rates as supported by numerous studies.^[25–27]^

More recently, some researchers used a genetic risk score, which adopts risk variants to predict CRC. Studies in Korea were based on 7 SNPs, including rs10795668, to develop a genetic risk score for prediction and had significant associations with CRC.^[28]^ Win et al^[29]^ observed that heterozygous and homozygous carriers of the G alleles for rs10795668 decreased CRC risk only among PMS2 mutation carriers. A meta-analysis of genetic associations between SNPs and colorectal cancer risk published by Hong et al,^[30]^ confirmed significant associations with a risk variant at rs6983267. In the study, subjects in the medium and high tertiles of CYP2A6 activity had an increased risk of colorectal cancer compared with subjects with low activity.^[31]^ In our study, in the case of CYP2A13, it is observed that individuals with blood type A have 21.60% with the Wild genotype, 17.33% with Heterozygous, and 22.22% with the Mutant genotype. For instance, a *P*-value of 0.151 for the Rh+ and Rh-blood group association in CYP2A13 indicates a relatively weak correlation, while a *P*-value of 0.100 for the association between the Wild genotype of CYP2A6 gene or cytochrome P450 2A6 enzyme (CYP2A6a) and blood groups suggests a slightly stronger relationship.

However, it is uncertain why this difference in survival is not obvious in regression and difference analyses. The conflicting nature of the findings of several studies was evident. Inconsistencies in endpoint assessment, retroactive data collection, and a variety of genetic origins might all be contributing factors to the differences. Finally, further clinical studies with longer durations of follow-up are required to be undertaken to accurately identify the link, with an emphasis on the genetic analysis of ABO and Rh blood antigens among colorectal cancer patients. Furthermore, disparities in findings across published research may be linked to differences in physiological consequences of ABO and Rh blood antigens between ethnicities, making multinational trials crucial in this field of research.

## 5. Limitations and strengths

It should be noted that the research conducted was hospital-based and may not be representative of the general population. Additionally, the possibility of additional contributing factors to CRC cannot be excluded. Despite these limitations, this research is the first of its type nationally, providing a comprehensive analysis of the ABO and RH blood group and CRC in indigenous populations, and being the first to investigate the potential survival and ABO and RH blood groups. The research’s prospective design minimized the risk of selection bias and the use of a detailed questionnaire at baseline allowed for controlling for any confounding factor. The high follow-up rate of the cohort members also reduced the possibility of bias due to the loss of follow-up for cancer incidence and mortality. It has been observed that due to limited number of cases and the potential failure to achieve statistical significance required for the study design may be one of the reasons for not observing significant differences.

## 6. Conclusion

In conclusion, the results of this study indicate that ABO and Rh blood groups are not correlated with the risk of CRC or the overall survival of CRC patients. Further clinical investigations are warranted to elucidate the exact connection between blood groups and survival rates, along with their potential significance for the prognosis of CRC patients.

## Acknowledgments

We are highly indebted to all the study participants who willingly participated in this study.

## Author contributions

**Formal analysis:** Tahseen Bilal Rather, Mohanad Diab.

**Investigation:** Gowhar Rashid.

**Methodology:** Gulzar A Bhat, Tahseen Bilal Rather.

**Project administration:** Syed Nisar Ahmad, Malik Tariq Rasool, Mudassar Syed.

**Resources:** Syed Nisar Ahmad, Malik Tariq Rasool, Farooq Ahmad Jan, Mudassar Syed.

**Software:** Mohanad Diab, Wael Hafez.

**Supervision:** Mudassar Syed.

**Validation:** Kulsum Akhtar, Mudassar Syed.

**Visualization:** Tahseen Bilal Rather, Kulsum Akhtar, Ishrat Parveiz, Mudassar Syed.

**Writing – original draft:** Gowhar Rashid.

**Writing – review & editing:** Gulzar A Bhat, Wael Hafez, Mudassar Syed.
